# Factors Associated with Emotion Regulation in Men with Internet Access Living in Brazil during the COVID-19 Pandemic

**DOI:** 10.3390/ijerph19073877

**Published:** 2022-03-24

**Authors:** Jules Ramon Brito Teixeira, Anderson Reis de Sousa, Emanuel Missias Silva Palma, Wanderson Carneiro Moreira, Thiago da Silva Santana, Nilo Manoel Pereira Vieira Barreto, Maciel Alves de Moura, Oscar Javier Vergara-Escobar, Oscar Yovani Fabián José, Gildásio Souza Pereira, Paulo Henrique Martins de Oliveira, Jacilene Santiago do Nascimento Trindade dos Santos, Emerson Lucas Silva Camargo, Tânia Maria de Araújo, Isabel Amélia Costa Mendes, Carla Aparecida Arena Ventura, Evanilda Souza de Santana Carvalho, Álvaro Francisco Lopes de Sousa

**Affiliations:** 1 Health Department, State University of Feira de Santana, Feira de Santana 44001-970, BA, Brazil; julesramon@gmail.com (J.R.B.T.); tssantana@uefs.br (T.d.S.S.); araujo.tania@uefs.br (T.M.d.A.); evasscarvalho@uefs.br (E.S.d.S.C.); 2 School of Nursing, Federal University of Bahia, Salvador 40231-300, BA, Brazil; nilo.manoel@ufba.br (N.M.P.V.B.); jacilenesnts@hotmail.com (J.S.d.N.T.d.S.); 3 Bahian School of Medicine and Public Health, Salvador 40231-300, BA, Brazil; emanuelmssilva@gmail.com; 4 School of Nursing, University of São Paulo, Sao Paulo 05403-000, SP, Brazil; wandersonm.wm@gmail.com; 5 Coordination of Health for Adolescents, Young People and Men, Technical Development Directorate, Health’s Secretary, Ananindeua 67130-600, PA, Brazil; 6 UniFTC University Center, Salvador 41741-590, BA, Brazil; maciel.123alves@hotmail.com; 7 School of Nursing, Juan N. Corpas University Foundation, Bogota 111321, Colombia; oscar.vergara@juanncorpas.edu.co; 8 Faculty of Nursing, Veracruz University, Minatitlán 96760, VE, Mexico; ofabian@uv.mx; 9 Jorge Amado University Center, Salvador 41680-400, BA, Brazil; gilpereiraintensive@gmail.com; 10 Multidisciplinary Institute in Health, Federal University of Bahia, Salvador 40231-300, BA, Brazil; paulohmo@hotmail.com; 11 Faculty of Psychology, University of Ribeirão Preto, Ribeirão Preto 11440-003, SP, Brazil; lucmrg0@gmail.com; 12 Ribeirão Preto School of Nursing, University of São Paulo, Ribeirão Preto 14040-902, SP, Brazil; iamendes@usp.br (I.A.C.M.); caaventura@gmail.com (C.A.A.V.); sousa.alvaromd@gmail.com (Á.F.L.d.S.); 13 Global Health and Tropical Medicine, Institute of Hygiene and Tropical Medicine, NOVA University of Lisbon, 1349-008 Lisbon, PT, Portugal

**Keywords:** pandemics, COVID-19, emotional regulation, men’s health, mental health

## Abstract

***Objective:*** to evaluate the factors associated with emotion regulation in men with internet access living in Brazil during the COVID-19 pandemic. ***Method:*** an epidemiological survey, conducted with 1015 men. An electronic form was applied containing sociodemographic and occupational characteristics, support and coping strategies, as well as emotional and behavioral aspects. Emotion regulation was assessed using the *Emotion Regulation Questionnaire*. ***Results:*** The prevalence values observed were 44.6% for Low Cognitive Reappraisal and of 47.1% for High Emotional Suppression. The following factors were identified as associated: (a) with Low Cognitive Reappraisal: being aged 30 years old or more, practicing physical activity, worrying about social distancing and having positive emotions and feelings; and (b) with High Emotional Suppression: being heterosexual, non-white race/skin color, having security support or public administration, not sanitizing food, worrying about lack of physical activity and not having negative emotions. ***Conclusion:*** the adoption of emotion regulation strategies was associated with individual, contextual and emotional/behavioral characteristics. Masculinity ideals seem to exert an influence on these relationships.

## 1. Introduction

The emergence of the COVID-19 (Coronavirus Disease 2019) pandemic and its rapid spread across countries resulted in difficulties in accessing adequate treatment and more accurate information related to the disease, which resulted in an outbreak of negative feelings and emotions, such as fear of helplessness and hopelessness among populations [1,2,3,4,5,6].

With regard to sex and gender, it is clear that such variables are important modifiers of mental health and behavior in normal times and during health crises [7]. It is understood that sex generally refers to a biological construct, while gender encompasses psychosocial variables that characterize women and men and their life contexts [8,9].

In this sense, in relation to the female biological sex, it is assumed that women with a cisgender gender identity may be more professionally exposed to the virus, as they represent the vast majority (about 70%) of health professionals, day care centers, teachers and service providers in stores and restaurants [10]. However, a pattern of predominance of male mortality in the COVID-19 pandemic has been identified in several countries, and repeated in Brazil, due to aspects such as hormonal and chromosomal function, presence of previous comorbidities and maintenance of health risk patterns, habits and lifestyles, as observed in smoking, related to the gender marker of masculinities [11]. In addition, there is evidence that men may be more vulnerable to mental health problems, such as high levels of emotional dysregulation [3,12].

Emotion regulation refers to the process of trying to influence emotional states experienced and/or expressed, one’s own and/or others’, whether deliberately or automatically, when it is perceived that the emotions felt are dysfunctional or inadequate in the context in which they are. That influence occurs through changes in the physiological, cognitive and behavioral components of the emotion. With this, the emotional experience can be adjusted in terms of type, intensity and duration [13].

The emotion regulation process is activated to attain two main goals: the first, and most prevalent, would be to improve well-being by trying to reduce the negative emotions and increase the positive ones; the second aims at experiencing or presenting emotions more adjusted to the context and that help to achieve satisfactory non-emotional results [14].

There are five sets of emotion regulation strategies, ranging from trying to approach, moving away from or modifying the situation that elicits the unwanted emotion (situation selection and modification strategies), to redirecting attention within a given situation or its thoughts (reorientation of attention), modifying the thoughts about the situation and changing its emotional meaning (cognitive modification) or even modifying the physiological, experimental and behavioral components of the emotion after its complete activation (response modulation) [13,15].

Cognitive modification strategies and response modulation strategies have been more widely investigated and represented by two main strategies, Cognitive Reappraisal and Emotional Suppression [16], respectively. There is empirical evidence that emotional suppression is often triggered early in the self-regulatory process, whereas cognitive reappraisal appears after the emotional episode [16]. In addition to that, suppression has been more related to harm in well-being, potentiating negative emotions and emotional exhaustion, while reappraisal is more related to improving well-being, in addition to decreasing the intensification of the negative emotions, suggesting a greater adaptive value of this last strategy [17,18,19,20].

The literature shows that some factors can influence the selection and implementation of emotion regulation strategies, from individual determinants (e.g., sex, age) to individual emotion (e.g., quality, intensity) [21,22,23]. In addition to that, intense negative emotions immediately trigger emotion suppression strategies, given the challenge of regulating this type of affect as it requires greater effort and emotional skill [22].

Considering that the COVID-19 pandemic has resulted in true psychological chaos for the world’s population and that epidemiological studies that have investigated sex and gender as moderators of mental health have been rare to date, it is essential that there is a situational diagnosis in specific groups, especially in the male population, so that care plans are designed to meet their real needs. Thus, this study aims to evaluate the factors associated with emotion regulation in men with internet access living in Brazil during the COVID-19 pandemic.

## 2. Method

### 2.1. Type of Study

A cross-sectional and analytical study conducted in a virtual environment with men living in Brazil. To ensure quality of the study, the *Reporting of Observational Studies in Epidemiology* (STROBE) statement was complied with. The research study took place between March and May 2020, during the critical period of social distancing determined by the Brazilian health authorities, due to the COVID-19 pandemic.

### 2.2. Population and Sample

For the sample calculation, we used as parameters: a population of 64,520,660 Brazilian men with internet access [24], expected prevalence of 50% considering that the outcome is unknown for this population group, confidence level of 95%, precision of 5%, power of 80%, study design effect of 2, and 20% addition for losses. We estimate a minimum sample size of 923 participants.

We recruited participants on digital social networks (Facebook, Instagram, WhatsApp) using the snowball technique [25]. It is a non-probabilistic sampling technique, conducted according to reference chains. In these chains, we initially recruited the first 25 eligible participants from each region of Brazil, who were called seeds, and they invited new participants from their network of contacts, who could be family, friends and acquaintances. Then, they invited new participants etc., until the estimated sample is minimally satisfactory. A total of 1015 men living in Brazil participated in the study.

### 2.3. Eligibility Criteria

The following inclusion criteria were adopted: being literate, having access to the Internet and being 18 years old or more. Tourists who were in the country at the time of data collection were excluded.

### 2.4. Data Collection Instruments and Procedures

Collection was carried out through an electronic form hosted on a free digital platform provided by *Google Forms*. The research participants were invited by sharing the link with the form via digital social networks (*Facebook*, *Instagram* and *WhatsApp*).

The form consisted of thematic blocks to assess the following: 1—Sociodemographic (sexual identity, age, race/skin color, schooling, monthly income, with whom they live) and occupational (work status and employment contract) characteristics; 2—Support and coping strategies for the pandemic (use of the health system, type of support during the pandemic and strategies to facilitate coping); 3—Emotional and behavioral aspects (reasons for concern, attitudes and needs during the pandemic and emotional regulation).

Sexual identity was dichotomized into heterosexual and non-heterosexual (homosexual, bisexual, transsexual, pansexual, asexual and others). Race/Skin color was self-reported and dichotomized into white and non-white (Asian, indigenous, brown and black). As for the work situation, men who had a formal contract or were statutory employees were considered as having formal contracts.

The emotions were self-reported and dichotomized into positive (love, trust, emotion, empathy) and negative (anxiety, stress, fear, insecurity, instability and boredom), requiring men to report on their experience regarding at least one of them. Similarly, the feelings were classified as positive (encouragement, motivation, optimism and tranquility) and negative (anxiety, impotence, pessimism and lack of motivation).

The needs experienced by men during the pandemic were analyzed according to Maslow’s Basic Human Needs Theory [26], being categorized as follows: (a) Safety needs (health and social care, medications, physical activity, public mobility, work/employment, access to alcohol gel, mask and hypochlorite); and (b) physiological needs (food, housing, provisions, groceries, clothing, personal and household hygiene products and others, drinking water, electricity, cooking gas, sex).

To assess emotional regulation, the *Emotion Regulation Questionnaire* (ERQ) was applied, as proposed by Gross and John [16] and validated for use in the Brazilian population [27,28], consisting of 10 items that assess two strategies used to regulate emotions: cognitive reappraisal (6 items) and emotional suppression (4 items). The answer options are organized on a *Likert*-type scale ranging from 1 (Strongly Disagree) to 7 (Strongly Agree). The scores were calculated by adding the points assigned to each subscale, with higher levels indicating predominant use of a given strategy [27]. The median was used to dichotomize the low and high levels for each strategy [29]. In the analyses, high cognitive reappraisal and low emotional suppression and exposure to low cognitive reappraisal and high emotional suppression were considered as reference categories.

### 2.5. Data Analysis Procedures

The *Stata* software, version 14.0, was used for data processing and treatment. Prevalence, prevalence ratios (PR) and their respective confidence intervals (95% CI) were estimated, as well as absolute and relative frequencies for the categorical variables and minimum and maximum values, mean and standard deviation (SD) for the quantitative variables with normal distribution. To assess the association between the independent variables and each of the outcomes (Cognitive Reappraisal and Emotional Suppression), *Pearson*’s Chi-square test was employed in the bivariate analysis, selecting for inclusion those that presented *p*-values ≤ 0.20 in the multivariate stage. In multivariate modeling, Poisson regression with robust variation was conducted, using the *backward* procedure, to define the useful subset of terms. All the variables that 5% presented statistical significance remained in the final model. To determine the best final model, the one with the lowest *Akaike* Information Criterion (AIC) value was selected. The statistical significance level of the tests was 5% (*p*-value ≤ 0.05).

### 2.6. Ethical Aspects

The study was approved by the Research Ethics Committee of the Federal University of Bahia, under protocol No. 4,087,611, in compliance with the national and international ethical principles for research involving human beings.

## 3. Results

A total of 1015 men participated in the study. Among the emotion regulation strategies, a mean of 31.2 points was identified (Min.: 6 and Max.: 42; SD: ±6.8) for Cognitive Reappraisal and of 16.3 points (Min.: 4 and Max.: 28; SD: ±5.4) for Emotional Suppression. The prevalence values observed were 44.6% for Low Cognitive Reassessment and 47.1% for High Emotional Suppression (data not presented in the tables).

There was predominance of non-heterosexuals (57.1%), aged 30 years old or more (58.8%) (Min.: 18 and Max.: 75; $\bar{X}$: 33.3 and SD: 10.4), non-white (64.2%), monthly income of three or more wages (56.2%), living with family members/friends (78.9%), workers (72.5%) and formal work contracts (65.6%). There was an association between low cognitive reappraisal and age group (*p*-value = 0.006), where men aged 30 years old or more increased by 22% the use prevalence of this regulatory strategy (PR: 1.22; 95% CI: 1.06–1.41) (Table 1).

In the bivariate analysis, sexual identity (*p*-value < 0.001), monthly income (*p*-value = 0.007) and not living alone were associated with high emotional suppression (*p*-value = 0.049). Non-heterosexuals (PR: 0.78; 95% CI: 0.68–0.88) and those with lower incomes (PR: 0.83; 95% CI: 0.73–0.95) presented reduced prevalence values of high emotional suppression (Table 1). Being heterosexual (PR: 1.29; 95% CI: 1.13–1.47) and having an income of three or more minimum wages (PR: 1.20; 95% CI: 1.05–1.36) increases the prevalence of high emotional suppression by 29% and 20%, respectively (data not presented in the tables).

Regarding the characteristics of COVID-19’s coping, non-exclusive use of the SUS predominated (62.5%), as well as support from family and friends (78.2%), with a smaller proportion seeking security services or public administration (19.7%) and health care (13.7%). Among the strategies to facilitate coping analyzed, practice of leisure activities prevailed (97.7%) and, to a lesser extent, work (49.3%) and physical (31.5%) activities. Among the attitudes, most adhered to hand hygiene (94.3%), social distancing (91.0%), use of alcohol gel (78.2%), food sanitation (62.8%) and of the home environment (65.1%) and, to a lesser extent, use of mask (23.6%). Regarding the reasons for concern, social contact distancing (69.7%) prevailed and, to a lesser extent, lack of physical activity (42.7%), the emotional situation (40.8%) and that of the loving relationship (13.4%) (Table 2).

In the bivariate analysis, there was an association between low cognitive reappraisal and physical activity as a strategy to facilitate coping (*p*-value < 0.001), attitude towards use of alcohol gel (*p*-value = 0.004) and concern with social contact distancing (*p*-value = 0.027). This concern increased the prevalence of low cognitive reappraisal by 18% (PR: 1.18; 95% CI: 1.01–1.37), and not practicing physical activity (PR: 0.79; 95% CI: 0.69–0.91) and not using alcohol gel (PR: 0.83; 95% CI: 0.73–0.95) reduced the prevalence values (Table 2). Practicing physical activity (PR: 1.27; 95% CI: 1.10–1.45) and not using alcohol gel (PR: 1.30; 95% CI: 1.07–1.57) increased the prevalence of low cognitive reappraisal by 27% and 30%, respectively (data not presented in the tables).

As for high emotional suppression, in the bivariate analysis, there was an association with the following variables: use of the SUS (*p*-value = 0.002), support from security services or public administration (*p*-value = 0.014) and from family/friends (*p*-value = 0.043), work activity as a facilitating strategy (*p*-value = 0.025), concern with the situation of the love relationship (*p*-value = 0.032) and emotional (*p*-value = 0.036). Not having support from the family and/or friends increased the prevalence of high emotional suppression by 30% (PR: 1.30; 95% CI: 1.02–1.65) and not engaging in any work activity as a coping strategy did so by 16% (PR: 1.16; 95% CI: 1.02–1.33). Exclusive use of the SUS (PR: 0.80; 95% CI: 0.69–0.92), not seeking security or public administration services (PR: 0.72; 95% CI: 0.57–0.92) and worrying about the situation of the love relationship (PR: 0.79; 95% CI: 0.62–0.99) and about the emotional situation (PR: 0.86; 95% CI: 0.75–0.99) were protective factors for high emotional suppression (Table 2). It was identified that non-exclusive use of the SUS (PR: 1.25; 95% CI: 1.08–1.45), not seeking security or public administration services (PR: 1.38; 95% CI: 1.09–1.75) and not worrying about the situation of the love relationship (PR: 1.26; 95% CI: 1.01–1.59) and about the emotional situation (PR: 1.16; 95% CI: 1.01–1.33) increases the prevalence of high emotional suppression by 25%, 38%, 25% and 16%, respectively (data not presented in the tables).

There was a lower proportion of experience of positive emotions (45.7%). There were reports of experiencing negative emotions (83.0%), positive feelings (59.7%) and negative feelings (50.1%) among men. Among the basic human needs, those related to security predominated (62.1%) and, to a lower extent, the physiological ones (32.0%) (Table 3).

In the bivariate analysis, an association of low cognitive reappraisal with the positive emotions (*p*-value < 0.001), positive feelings (*p*-value < 0.001) and negative feelings (*p*-value = 0.041) variables was identified. Not experiencing positive emotions (PR: 0.71; 95% CI: 0.62–0.82) and feelings (PR: 0.66; 95% CI: 0.56–0.77) and negative feelings (PR: 0.87; 95% CI: 0.76–0.99) reduced the prevalence of low cognitive reappraisal (Table 3). Positive emotions (PR: 1.40; 95% CI: 1.22–1.60) increased this prevalence by 40%, positive feelings (PR: 1.52; 95% CI: 1.31–1.78) by 52% and negative feelings (PR: 1.15; 95% CI: 1.01–1.32) did so by 15% (data not presented in the tables). High emotional suppression was associated with the negative emotion’s variable (*p*-value = 0.024), in which experiencing them reduced the prevalence of this regulatory strategy (Table 3). Experiencing negative emotions (PR: 1.21; 95% CI: 1.03–1.41) increased the prevalence of high emotional suppression by 21% (data not presented in the tables).

In the multivariate analysis (Model 1), the prevalence of low cognitive reappraisal remained associated with the age group of 30 years old or more (PR: 1.24; 95% CI: 1.08–1.44) and with being concerned with social contact distancing (PR: 1.17; 95% CI: 1.01–1.35). Practicing physical activity as a strategy to facilitate coping (PR: 0.83; 95% CI: 0.72–0.96) and not experiencing positive emotions (PR: 0.81; 95% CI: 0.70–0.94) and feelings (PR: 0.72; 95% CI: 0.61–0.85) remained reducing this prevalence, with an increased association effect. Adjusting for the age, schooling, race/skin color, income and work relationship variables (Model 2), in addition to the variables identified in the unadjusted model, it was verified that worrying about the emotional situation reduced the prevalence of low cognitive reappraisal (PR: 0.83; 95% CI: 0.70–0.98) (Table 4).

There was an association and increase in the prevalence of high emotional suppression in non-white race/skin color (PR: 1.36; 95% CI: 1.06–1.75), attitude of not sanitizing food (PR: 1.31; 95% CI: 1.05–1.64) and concern about lack of physical activity (PR: 1.37; 95% CI: 1.10–1.72), which began to have a statistically significant association in the multivariate model. The following remained associated, reducing the prevalence of high emotional suppression: non-heterosexual sexual identity (PR: 0.78; 95% CI: 0.62–0.97), support of security services or public administration for coping (PR: 0.68; 95% CI: 0.54–0.85) and experiencing negative emotions (PR: 0.71; 95% CI: 0.55–0.93) (Table 4).

## 4. Discussion

Concern for the mental health of the population intensifies during a serious social crisis. The COVID-19 pandemic can be described as one of these crises, which has been characterized as one of the largest international public health problems in recent decades, having affected virtually the entire planet, according to the World Health Organization [30]. Such an event also causes psychological repercussions in specific groups, such as men at various intensity levels, interfering in the type of emotion regulation strategy used. A study that reviewed cases of patients with COVID-19 in Beijing found differences in sex and gender and found that men tended to be more severe than women, died 2.4 times more than women, and the role of gender in mortality by COVID-19 was observed, indicating that men with COVID-19 are at greater risk of worse outcomes and death, regardless of age [31]. A similar scenario was identified in a study carried out in Italy with 1175 cases of patients who had COVID-19, and recommended directing attention to the gender variable in the interpretation of data related to COVID-19, as a way to support health professionals in decision making.

Specifically in relation to the mental health situation, the findings in the literature, such as a study carried out in Brazil, indicated that older women perceived themselves to be more vulnerable to the pandemic than compared to men [32]. In Spain, scientific findings showed that although women had higher scores on psychological variables, gender differences disappeared with the length of the pandemic, in which women showed a significant improvement in psychological measures than men [33].

In this sense, it is important to understand how the individual and contextual characteristics are capable of influencing the type of emotion regulation strategy adopted by men in the COVID-19 pandemic context. While some tend to invest in more adaptive strategies, such as cognitive reappraisal, others tend to more non-adaptive strategies, such as suppression.

The results corroborate a study that identified that heterosexual men had higher levels of emotional suppression [34]. This relationship is partially due to how men are socialized not to express their emotions in order to meet expectations and social rules related to a strong internalized male ideology [34]. Such ideology can operate through a set of cognitive factors that restrict emotional expressiveness, especially in contexts involving other people. Another study presents those individuals who share other people’s negative emotional expressions on social media are likely to be affected by the negative affect contagion [35] and suggest the necessity to conduct social media-based health communication interventions to mitigate the social media-wide negative affect contagion if lockdown policies related to highly infectious diseases are initiated.

It is also due to a greater tendency of these men to inhibit their emotions when living with other people. The literature has pointed out the deleterious effects of the excessive use of emotional suppression, associating it with the development of psychopathologies and aggressive behavior [19,36]. Aspects related to the construction of masculinities, especially the hegemonic models of normative gender standardization [37,38,39], can weave an influence relationship on male emotional suppression, as was the case with the group of heterosexual men. For these reasons, the markers of masculinities need to be considered and deepened in order to locate the most relevant elements in the conduction of men to dull emotions and feelings [40], even in critical and complex events such as a pandemic [40,41,42].

It was evidenced that older men presented higher prevalence of low cognitive reappraisal. In relation to this, certain sense of emotional control can distinguish how different groups of people (e.g., young men vs. older men) experience emotions. Cognitive reappraisal is a background-focused emotional regulation strategy [43], and is therefore strongly influenced by the ideals of masculinity consolidated in men’s lives with advancing age. This problem may occur for the reasons that men have belatedly perceived vulnerabilities, have been slow to recognize the COVID-19 pandemic as a public health problem and cause psychological and behavioral damage, as well as have resisted restrictive measures of prevention and control of COVID-19, is what an analysis carried out in eight countries indicates [44]. Added to this is the way men dealt with emotions, suppressing them, being worthy of attention [45].

The naturalization of violent behavior since childhood and the experience of negative emotions and feelings [3], in addition to castrating, punishing, poignant and blunted, can be evoked for the understanding of this finding, as men may avoid cognitive reappraisal for fear of showing weakness in their actions in front of other men and society in general, responding to stimuli with impulsiveness and less planning of their reactions. For example, in the face of an insult or reprimand, men may tend to potentiate anger, blame the other and react with violence, even with previous experiences that would lead them to resignify this situation to produce new ways of thinking and acting [41]. Thus, in the COVID-19 pandemic context, much has been reported in the media about men’s violent reactions in the face of health interventions [44], such as restrictive measures regarding social contact and mandatory use of masks to enter or remain in certain places, which endorses low cognitive reappraisal in this group [46]. In the same way, other study shows that difficulties in regulating emotions and aggression may exacerbate risky driving behaviors, then deficits in cognitive inhibition and attentional bias toward negative emotional stimuli can increase errors and aggression is a significant predictive factor for violations.

As identified in this study, there is diverse evidence of increased adoption of high emotional suppression in non-white ethnic minorities, especially among black-skinned men. Since adolescence, black-skinned boys (extending to Latinos, Hispanics, and indigenous people) are taught to react coldly and indifferently as a way to fight against a sense of disrespect and discrimination, suppressing their emotions [47]. This emotion regulation reverberates in adulthood, in addition to being intensified. Moreover, this emotional suppression can also be associated with fear of punishment and criminalization by society, translating into a need for emotional hypervigilance and configuring itself as a survival strategy [47]. Thus, despite protecting these ethnic groups from violent attacks, high emotional suppression increases the risks of impacts on the health of these men, especially in relation to mental and cardiovascular health [47,48].

In Brazil, the search for security support or public administration is closely related to the need/difficulty of access to the respective services offered in the community. Thus, the results of this study about the relationship between not seeking support from these care spheres and high emotional suppression can be explained by some factors: (a) the increase in fear and anguish of being contaminated by COVID-19 when infringing the social distancing measure and entering these public agencies noticeable characterized by agglomerations, especially by people with some degree of illness; (b) the tension generated by the inoperability and irresoluteness of public administrative and security services can discourage men from seeking this support, as their needs may not be met; (c) hostility or aggressiveness are constant in the care provided in public services, especially those of the SUS; (d) cultural and institutional barriers distance men from the public services, because they want to have their needs met immediately, without facing long waiting lines: relying on these services generates stress, anxiety and emotional uncontrollability [3,49,50]. Thus, these circumstances require men to better monitor and suppress their emotions to limit a behavior that would be emotionally expressive aroused by factors related to the need for support offered by public administration and security services.

It was evidenced that those who were not practicing physical activity during the COVID-19 pandemic had higher prevalence of high cognitive reappraisal. In addition to that, men who worried about lack of physical activity had high levels of emotional suppression. Cognitive reappraisal allows men to adopt a more subjective way of dealing with the fact of not practicing physical activity, even knowing its health benefits, transforming negative emotions into positive responses from the mind [51]. In conflict situations, such as this pandemic context experienced, high emotional suppression is even more unadaptive [52] and inhibits men from expressing their true emotions; therefore, the distress resulting from the concern with lack of physical activity can be even greater than that mentioned.

In Brazil, in 2020, with the Public Health Emergency of National Importance (*Emergência de Saúde Pública de Importância Nacional*, ESPIN) decree, through Law No. 13,979 [53,54] and Ordinance No. 188, the Ministry of Health recommended the suspension of environments that promoted agglomeration of people due to the potential risk of COVID-19 transmission, such as gyms for physical activity [55]. The guidance was that people should choose to exercise in their homes and outdoors rather than indoors. However, many Brazilian states and municipalities decreed the mandatory closure of gyms during the first months of the pandemic [56], given the increase in the number of cases. Thus, many people stopped practicing these activities as a result of not considering having the necessary structure at their homes to practice the exercises or for being discouraged by the chaos experienced in the pandemic context.

However, home-based physical activity should be strongly encouraged, as it is a viable and important proposal for providing immediate and long-term health benefits, especially for vulnerable groups and/or during emergency periods of restricted social contact, as experienced during the COVID-19 pandemic [57].

High emotional suppression was associated in men who did not sanitize food. Household activities such as home care, children and eating habits have always been a role socially attributed to women, which may have generated discomfort, increased stress and emotional instability in men when dealing with adaptations of their routine by staying in the home space for longer periods of time [3,41]. For men, possibly, assuming this responsibility of sanitizing food after shopping or when arriving home, a necessary measure to contain virus transmission, may imply the perception of performing a function that they believe to be feminine. High emotional suppression can be due to the fact that they know it is a necessary activity, but that the ideals of masculinity based on machismo make them associate it with loss of virility and power in front of women, family, and society in general [40].

An association was identified between worrying about social contact distancing and low cognitive reappraisal. Restriction of social contact can result in increased anxiety and stress, and low cognitive reappraisal can accentuate this distressful situation. Thus, a high level of cognitive reappraisal provides an internal resource to reduce anxiety and stress [58]. Cognitive reappraisal is a skill that can be trained and could be utilized to buffer the effect of general stress on individuals’ wellbeing because the use of cognitive reappraisal to regulate emotions was associated with greater resilience (i.e., feelings of hope and resourcefulness, and ability to seek social support and enjoyable activities).

Having concerns about the emotional situation was associated with higher levels of cognitive reappraisal, but only after adjusting the model for age, schooling, race/skin color, monthly income and employment status. Considering that cognitive reappraisal is an adaptive strategy through which men can modulate their reactions in order to produce positive emotions in the face of a so-called bad situation, it is inferable that they are being able to externalize this concern with the emotional situation. In addition to that, differences in schooling and income can be associated with other confounding social variables, such as race/skin color and employment status, influencing greater access to information and health services, which, together, enable men to experience a context in which the expression of emotions and prior self-evaluation of actions are more valued.

Men who experienced positive emotions and feelings had lower levels of cognitive reappraisal. This result is apparently controversial and requires caution in its interpretation, as cognitive reappraisal is associated with improved well-being and with the experience of positive emotions and feelings [18,19,20]. There is much evidence that positive emotions and feelings, such as optimism, can increase flexible cognition, trigger intrinsic motivation, invigorate self-confidence, and stimulate creative thinking and behavior [40,59,60,61].

In an also unexpected way, not experiencing negative emotions was associated with high emotional suppression, and it is known that adoption of this strategy provides an ineffective regulation of stressful stimuli, causing harms to general well-being and potentiating negative emotions and feelings [18,19,20]. Thus, it is necessary to explore this association longitudinally in order to identify potential mediators of the effect observed.

Understanding factors associated with emotion regulation and its repercussions in the face of a public health crisis such as the one caused by the COVID-19 pandemic is important to prepare health professionals, the general population and specific groups not only for this moment, but especially for the post-pandemic period. This is because it is necessary to implement control strategies and warn the population about immediate and continued risks, as adherence to preventive measures will depend on how people perceive this threat, in addition to reversing long-term sequelae with measures that can help reduce or prevent future psychiatric and psychological problems [62]. It is therefore recommended to reduce doubtful information, especially if it can generate symptoms related to anxiety and stress in the short- and long-term, as well as to provide assistance and care to those who have been psychologically affected by the COVID-19 pandemic [63].

Despite progressive resumption of the daily routine, after the decline in the number of new cases and the decrease in community transmission, a series of consequences of the pandemic demand medium and long periods of time to be reversed, such as: stress, anxiety, depression, specific phobias, avoidance, compulsive behavior, physical symptoms and impairments in social functioning [2].

In this sense, it is fundamental to develop public policies to promote mental health since, due to the long-term sequelae, it is expected that the demand for mental health care by patients diagnosed with COVID-19, by their family members and by specific groups such as men, will tend to increase, due to the simultaneity and speed of the emergence of confirmed cases of the disease. This contributes to symptoms and mental disorders being triggered by the possibility of death, transmission and exposure to the virus, as well as by the impact of all the changes in social functioning resulting from the pandemic [59,60], as well as in other previous contexts [61,62,63,64,65].

### Study Limitations

The diverse evidence found is relevant in the field of human health and public health in general, but the study has limitations that must be considered. The impossibility of adopting random recruitment mechanisms for the participants, due to the pandemic context, precludes generalization of the results. Internet access is unequal in Brazil, allowing men who have better purchasing power or who live in more urbanized places to have access to the electronic form. The cross-sectional design exerted an impact on the determination of cause and effect, limiting interpretation of the results and establishment of inferences.

## 5. Conclusions

The adoption of emotional regulation strategies by Brazilian men was associated with individual (sexual identity, age, race/skin color), contextual (support and coping strategies) and emotional/behavioral (concerns, attitudes and needs) characteristics related to the COVID-19 pandemic. Masculinity ideals seem to exert an influence on these relationships. It is necessary to encourage a redefinition of the life experiences so that emotional regulation strategies are protective factors for men’s mental health.

## Figures and Tables

**Table 1:** Distribution of the prevalence of Low Cognitive Reappraisal and High Emotional Suppression, according to sociodemographic and occupational characteristics, among Brazilian men in the COVID-19 pandemic context. Brazil, 2020 (*N* = 1015).

Variables	%	Low Cognitive Reappraisal					High Emotional Suppression				
		*n*	P	*p*-Value *	PR	95% CI	*N*	P	*p*-Value *	PR	95% CI
**Sociodemographic characteristics**											
**Sexual identity**											
Heterosexual (435)	42.9	198	45.5	0.623	1.00	-	235	54.0	<0.001	1.00	-
Non-heterosexual (580)	57.1	255	44.0		0.97	0.84–1.11	243	41.9		0.78	0.68–0.88
**Age group**											
18–29 years old (418)	41.2	165	39.5	0.006	1.00	-	202	48.3	0.511	1.00	-
30 years old or more (597)	58.8	288	48.2		1.22	1.06–1.41	276	46.2		0.96	0.84–1.09
**Schooling**											
Elementary School (66)	6.5	36	54.5	0.094	1.24	0.98–1.57	35	46.7	0.318	1.14	0.90–1.44
High School/Higher Education (949)	93.5	417	43.9		1.00	-	443	53.0		1.00	-
**Ethnicity**											
White (363)	35.8	172	47.4	0.188	1.00	-	159	43.8	0.117	1.00	-
Non-white (652)	64.2	281	43.1		0.91	0.79–1.05	319	48.9		1.12	0.97–1.29
Monthly income **											
Up to 2 wages (445)	43.8	185	41.6	0.083	1.13	0.98–1.30	231	51.9	0.007	0.83	0.73–0.95
3 or more wages (570)	56.2	268	59.2		1.00	-	247	43.3		1.00	-
**Living alone**											
No (801)	78.9	357	44.6	0.939	1.00	-	390	48.7	0.049	1.00	-
Yes (214)	21.1	96	44.9		1.01	0.85–1.19	88	41.1		0.84	0.71–1.01
**Occupational characteristics**											
**Work situation**											
Working (736)	72.5	336	45.7	0.288	1.00	-	339	46.1	0.284	1.00	-
Not working (279)	27.5	117	41.9		0.92	0.78–1.08	139	49.8		1.08	0.94–1.25
**Employment contract (N = 736)**											
Formal (483)	65.6	231	47.8	0.102	1.00	-	225	46.6	0.693	1.00	-
Informal (105)	34.4	105	41.5		0.87	0.73–1.03	114	45.1		0.97	0.82–1.14

P: Prevalence; PR: Prevalence ratio; 95%CI: 95% confidence interval. * *p*-Value obtained by *Pearson’s* chi-square test. ** Minimum wage in force in Brazil at the time of data collection: R$ 1.045,00.

**Table 2:** Distribution of the prevalence of Low Cognitive Reappraisal and High Emotional Suppression, according to characteristics related to coping and reasons for concern, among Brazilian men in the COVID-19 pandemic context. Brazil, 2020 (*N* = 1015).

Variables	%	Low Cognitive Reappraisal					High Emotional Suppression				
		*n*	P	*p*-Value *	PR	95% CI	*N*	P	*p*-Value *	PR	95% CI
**Coping characteristics**											
**Use of the SUS (N = 978)**											
Not exclusive (611)	62.5	282	46.2	0.202	1.00	-	309	50.6	0.002	1.00	-
Exclusive (367)	37.5	154	42.0		0.91	0.78–1.05	148	40.3		0.80	0.69–0.92
**Support to cope with the pandemic: Health Care (386)**											
Yes (53)	13.7	29	54.7	0.269	1.00	-	24	45.3	0.845	1.00	-
No (333)	86.3	155	46.5		0.85	0.65–1.12	146	43.8		0.97	0.70–1.33
**Support to cope with the pandemic: Public Security or Administration (386)**											
Yes (76)	19.7	40	52.6	0.334	1.00	-	43	56.6	0.014	1.00	-
No (310)	80.3	144	46.5		0.88	0.69–1.13	127	41.0		0.72	0.57–0.92
**Support to cope with the pandemic: family/friends (386)**											
Yes (301)	78.2	138	45.8	0.148	1.00	-	124	41.2	0.043	1.00	-
No (84)	21.8	46	54.8		1.19	0.95–1.50	45	53.6		1.30	1.02–1.65
**Strategy to facilitate coping: leisure activity (N = 994)**											
Yes (971)	97.7	438	45.1	0.325	1.00	-	460	47.4	0.711	1.00	-
No (23)	2.3	8	34.8		1.01	0.86–1.18	10	43.5		0.95	0.82–1.11
**Strategy to facilitate coping: physical activity (N = 994)**											
Yes (313)	31.5	438	52.4	0.001	1.00	-	137	43.8	0.133	1.00	-
No (681)	68.5	8	41.4		0.79	0.69–0.91	333	48.9		1.11	0.96–1.29
**Strategy to facilitate coping: work activity (N = 994)**											
Yes (490)	49.3	222	45.3	0.785	1.00	-	214	43.7	0.025	1.00	-
No (504)	50.7	224	44.4		0.98	0.85–1.13	256	50.8		1.16	1.02–1.33
**Attitudes: Social distancing**											
Yes (924)	91.0	409	44.3	0.454	1.00	-	428	46.3	0.116	1.00	-
No (91)	9.0	44	48.4		1.09	0.87–1.37	50	54.9		1.19	0.97–1.45
**Attitudes: Hand hygiene**											
Yes (957)	94.3	433	45.2	0.109	1.00	-	450	47.0	0.853	1.00	-
No (58)	5.7	20	34.5		0.76	0.53–1.09	28	48.3		1.03	0.78–1.35
**Attitudes: Food sanitation**											
Yes (637)	62.8	296	46.5	0.126	1.00	-	287	45.1	0.091	1.00	-
No (378)	37.2	157	41.5		0.89	0.77–1.03	191	50.5		1.12	0.98–1.28
**Attitudes: Home environment sanitation**											
Yes (661)	65.1	309	46.7	0.064	1.00	-	306	46.3	0.485	1.00	-
No (354)	34.9	144	40.7		0.87	0.75–1.01	172	48.6		1.05	0.92–1.20
**Attitudes: Use of alcohol gel**											
Yes (794)	78.2	373	47.0	0.004	1.00	-	381	48.0	0.281	1.00	-
No (221)	21.8	80	36.2		0.77	0.64–0.93	97	43.9		0.91	0.77–1.08
**Attitudes: Use of mask**											
Yes (240)	23.6	110	45.8	0.668	1.00	-	118	49.2	0.462	1.00	-
No (775)	76.4	343	44.3		0.97	0.82–1.13	360	46.5		0.94	0.81–1.10
**Reasons for concern:**											
**Concern about social contact distancing (N = 977)**											
No (394)	40.3	157	39.8	0.027	1.00	-	198	50.3	0.082	1.00	-
Yes (583)	59.7	274	47.0		1.18	1.01–1.37	260	44.6		0.89	0.78–1.01
**Concern about lack of physical activity (N = 977)**											
No (560)	45.2	253	42.7	0.438	1.00	-	249	44.5	0.080	1.00	-
Yes (417)	42.7	178	44.1		0.94	0.82–1.09	209	50.1		1.13	0.99–1.29
**Concern about the situation of the love relationship (N = 977)**											
No (846)	86.6	379	44.8	0.274	1.00	-	408	48.2	0.032	1.00	-
Yes (131)	13.4	52	39.7		0.89	0.71–1.11	50	38.2		0.79	0.62–0.99
**Concern about the emotional situation (N = 977)**											
No (578)	59.2	267	46.2	0.115	1.00	-	287	49.7	0.036	1.00	-
Yes (399)	40.8	164	411		0.89	0.77–1.03	171	42.9		0.86	0.75–0.99

P: prevalence; PR: prevalence ratio; 95% CI: 95% confidence interval; SUS: *Sistema Único de Saúde*. * *p*-value obtained by means of Pearson’s Chi-Square test.

**Table 3:** Distribution of the prevalence of Low Cognitive Reappraisal and High Emotional Suppression among Brazilian men, according to emotions, feelings and needs experienced in the COVID-19 pandemic context. Brazil, 2020 (*N* = 1015).

		*n*	P	*p*-Value *	PR	95% CI	*N*	P	*p*-Value *	PR	95% CI
**Positive emotions**											
Yes (464)	45.7	245	52.8	<0.001	1.00	-	210	45.3	0.282	1.00	-
No (551)	54.3	208	37.7		0.71	0.62–0.82	283	48.6		1.07	0.94–1.22
**Negative emotions**											
No (173)	17.0	86	49.7	0.140	1.00	-	95	54.9	0.024	1.00	-
Yes (842)	83.0	367	43.6		0.88	0.74–1.04	383	45.5		0.83	0.71–0.97
**Positive feelings**											
Yes (606)	59.7	314	51.8	<0.001	1.00	-	272	44.9	0.086	1.00	-
No (409)	40.3	139	34.0		0.66	0.56–0.77	206	50.4		1.12	0.98–1.28
**Negative feelings**											
No (506)	49.9	242	47.8	0.041	1.00	-	245	48.4	0.399	1.00	-
Yes (509)	50.1	211	41.5		0.87	0.76–0.99	233	45.8		0.95	0.83–1.08
**Safety needs**											
No (385)	37.9	162	42.1	0.201	1.00	-	385	37.9	0.181	1.00	-
Yes (630)	62.1	291	46.2		1.10	0.95–1.27	630	62.1		1.10	0.96–1.26
**Physiological needs**											
No (690)	68.0	298	43.2	0.178	1.00	-	318	46.1	0.349	1.00	-
Yes (325)	32.0	155	47.7		1.10	0.96–1.27	160	49.2		1.07	0.93–1.22

P: prevalence; PR: prevalence ratio; 95% CI: 95% confidence interval. * *p*-value obtained by means of Pearson’s Chi-Square test.

**Table 4:** Prevalence ratios and confidence intervals (95%) of the factors associated with Low Cognitive Reappraisal in men in the COVID-19 context. Brazil, 2020.

Variables	Low Cognitive Reappraisal				High Emotional Suppression	
	Model 1		Model 2 ^a^			
	PR	95% CI	PR	95% CI	PR	95% CI
**Sexual identity**						
Heterosexual					1.00	-
Non-heterosexual					0.78	0.62–0.97
**Age group**						
18–29 years old	1.00	-				
30 years old or more	1.24	1.08–1.44				
**Ethnicity**						
White					1.00	-
Non-white					1.36	1.06–1.75
**Support to cope with the pandemic: Public Security or Administration**						
Yes					1.00	-
No					0.68	0.54–0.85
**Strategy to facilitate coping: physical activity**						
Yes	1.00	-	1.00	-		
No	0.83	0.72–0.96	0.80	0.69–0.95		
**Attitudes: Food sanitation**						
Yes					1.00	-
No					1.31	1.05–1.64
**Concern about social contact distancing**						
No	1.00	-	1.00	-		
Yes	1.17	1.01–1.35	1.22	1.03–1.44		
**Concern about lack of physical activity**						
No					1.00	-
Yes					1.37	1.10–1.72
**Concern about the emotional situation**						
No			1.00	-		
Yes			0.83	0.70–0.98		
**Positive emotions**						
Yes	1.00	-	1.00	-		
No	0.81	0.70–0.94	0.79	0.66–0.93		
**Negative emotions**						
No					1.00	-
Yes					0.71	0.55–0.93
**Positive feelings**						
Yes	1.00	-	1.00	-		
No	0.72	0.61–0.85	0.78	0.65–0.95		
AIC	1.5882		1.6272		1.5916	

PR: prevalence ratio; 95% CI: 95% confidence interval. ^a^ Model adjusted for age, schooling, race, income and employment contract.

## Data Availability

The dataset generated during the current study are not publicly available but are available from the corresponding author on reasonable request.
